# Prevalence and Risk Factors of Deep Vein Thrombosis Among Adult Surgical Patients in Aseer Central Hospital, Saudi Arabia

**DOI:** 10.7759/cureus.47856

**Published:** 2023-10-28

**Authors:** Abdulrahim S Alamri, Meshal S Alamri, Faiza Al-Qahatani, Abdulrahman S Alamri, Abdulaziz M Alghuthaymi, Ali M Alamri, Hadi M Albalhsn, Ammar N Alamri

**Affiliations:** 1 College of Medicine, University of Bisha, Bisha, SAU; 2 General Medicine and Surgery, Private Practice, Abha, SAU; 3 Nursing, Male Neurology Ward, Aseer Central Hospital, Abha, SAU; 4 College of Medicine, King Khalid University, Abha, SAU; 5 Medicine and Surgery, University of Bisha, Bisha, SAU

**Keywords:** thromboprophylaxis, venous stasis, surgical patients, saphaneous veins, blood clots, aseer region, kingdom of saudi arabia (ksa), surgery, deep vein thrombosis (dvt)

## Abstract

Introduction: Deep vein thrombosis (DVT) is a medical disorder that arises when a coagulation of blood forms in a deep vein, entirely or partially blocking veins, and commonly affects the lower limb. The occurrence is fairly common worldwide and it is said to increase with age, with males being at a higher risk than females.

Objective: This study aims to determine the prevalence and risk factors of DVT among adult surgical patients in Aseer Central Hospital in the Aseer Region of Saudi Arabia.

Methods: This is a cross-sectional study involving 602 adult surgical patients hospitalized in the Aseer Central Hospital. Self-administered questionnaires were used to collect data from the respondents, and the data collected were analyzed using IBM SPSS Statistics for Windows, Version 24.0 (Released 2016; IBM Corp., Armonk, New York, United States). Statistical tests of association were used among the categorical variables. Association between variables was considered significant when p-value was less than 0.05. Binary logistic regression was performed to eliminate the effect of confounders in determining the risk factors for developing DVT among the respondents.

Results: The questionnaire response rate was 100%, with the mean age of the respondents being 44.2 ± 19.7 years. The prevalence of DVT was 7% (n=42). Obesity with adjusted OR (aOR) 17.9 (95%CI =5.39-59.18), hypertension with aOR 0.3 (95%CI =0.08-1.03), ischemic heart disease with aOR4.5 (95%CI =1.18-16.83), and orthopedics aOR 0.1 (95%CI=0.013-.240) were found to be independent risk factors for developing DVT among the respondents (p-value <=0.05). Other variables like diabetes, contraception, and pregnancy were not statistically associated with the development of DVT (p-value> 0.05) in these respondents.

Conclusion: The findings of this study indicated a significantly low prevalence in comparison to Saudi Arabian research. Key risk factors included obesity (18x higher risk), ischemic heart disease, and hypertension. Surgery location, orthopedic cast, and Doppler ultrasound also influenced risk, while age and sex weren't significant predictors.

## Introduction

Deep vein thrombosis (DVT) is the formation of one or more blood clots in one of the body's large veins, most commonly in the lower limbs. The key mechanisms of DVT development are venous stasis, hypercoagulability, and endothelial Injuries. The great saphenous veins are more likely to be affected by this illness, but there are other circumstances in which the blood clot may go to the lungs and cause pulmonary embolism (PE) [1]. DVT is considered relatively uncommon among surgical patients but early diagnosis is crucial. It often affects PE, which can occasionally cause severe morbidity and impair quality of life or even be fatal. Findings indicate that 50-80% of PE cases are associated with DVT, accounting for nearly half of all DVT cases that go untreated [2].

The main risk factors for DVT are immobility, major surgery, obesity, old age, and pregnancy [3]. Some factors contributing to venous stasis in surgery patients include postoperative bed rest, venous compression, lack of muscle tone, and horizontal vernal decubitus [3]. Significant venous intimal damage can also develop while a patient is undergoing surgery. About 15% of people undergoing posterior spinal surgery are vulnerable to the consequences of DVT, and these risk factors are particularly prevalent in patients without prophylaxis and those with degenerative spine conditions. Although several treatments are available for DVT, it is particularly challenging for doctors to use thromboprophylaxis in patients after spine surgery due to the lack of proof of the health consequences [4]. Additionally, although research suggests that not treating DVT may result in additional health issues, other findings imply that overtreating the illness may increase bleeding rates, including gastrointestinal and cerebral hemorrhages.

According to estimates, the incidence is 1/1000 people worldwide each year and increases with age [5]. The incidence varies considerably in different studies [6,7]. This variation underscores the essence of a focused regional study. The main objective of this study is to identify the prevalence of DVT and its risk factors among surgical patients in the southern region of Saudi Arabia with a special focus on Aseer Central Hospital, Saudi Arabia. Understanding the prevalence and risk factors will be imperative in post-operative care and preventing complications. 

## Materials and methods

This was a cross-sectional hospital-based study conducted in Aseer Central Hospital in the Aseer Region of Saudi Arabia from January 2023 to August 2023. The Aseer Central Hospital Institutional Review Board approved the study (approval number: REC-11-08-2022). Informed consent and confidentiality were some of the ethical considerations. The respondents were assured that they had the right to withdraw consent and abandon the process at any point during the survey.

Sample size

The estimated sample size was 602 and determined using the formula $ss = \frac{(Z^{2}pq)}{c^{2}}$, where

ss = sample size, Z = 1.96, p = 0.5, q = (1-p) = 0.5, and c = sampling error of 4%.

The subjects were selected through convenience sampling techniques as it was the most appropriate technique to sample the surgical patients.

Inclusion and exclusion criteria

This study included adult surgical patients who were hospitalized at Aseer Central Hospital and were stable and willing to participate in the study. The exclusion criteria were critically ill patients and patients who were receiving ambulatory care. Also since informed consent was an imperative ethical consideration, we excluded the patients that were unwilling to participate.

Data collection methods

Data were collected through a validated questionnaire and issued to the target population. Self-administered questionnaires were distributed to surgical patients in Aseer Central Hospital during the study period. Preoperative nurses based in the hospital facilitated ease of access to the patients. The questionnaire was made up of three key sections. The first section involved social demographic information, the second section involved items on risk factors and the third section had items that were complimented by available health records. The questionnaire originally in English was translated into Arabic for the purpose of data collection. We developed the survey using Google Forms (Google LLC, Mountain View, California, United States) and it was self-administered by stable surgical patients.

Data analysis

The data collected were analyzed using IBM SPSS Statistics for Windows, Version 24.0 (Released 2016; IBM Corp., Armonk, New York, United States). Descriptive statistics were used to obtain frequency and percentages. Inferential statistics were also obtained using the Chi-square test. Bivariate logistic regression analysis was used to compute important statistical associations. Statistical significance was established when the data achieved a p-value of ≤ 0.05

## Results

The total number of questionnaires administered was 602 giving a response rate of 100%. The age of the respondents ranged from 15 years to 96 years. The mean age of the patients was 44.4 ±19.7 years. The majority were males, forming nearly two-thirds of the population (66.2%) while 33.7% were females. A total of 353 (58.7%) were below the age of 50 years. The educational level (Table 1) varied with about 323 (53.7%) having a high school certificate. The majority of the patients (n=394; 63.3%) were married. All the respondents came into the hospital through various units of the surgical department.

**Table 1:** Socio-demographic characteristics

Variables	Socio-demographic characteristics	Frequency, N=602	Percentages (%)
Sex	Female	203	33.7
	Male	399	66.2
Age (years)	18-30	202	33.6
	31-50	151	25.1
	Above 50	249	41.4
Marital status	Single	193	32.1
	Married	394	65.5
	Divorced	5	0.8
	Widowed	10	1.7
Educational level	Elementary school	81	13.4
	High school	323	53.7
	Bachelor’s degree	181	30.1
	Higher education	17	2.8
Site of surgery	Pelvis	22	3.7
	Head	16	2.7
	Lower limb (the thigh or the leg or the foot)	248	41.2
	Neck	12	1.9
	Trunk (the chest, the abdomen, and the back)	268	44.5
	Upper limb (the arm or the forearm or the hand)	36	5.9

The most common site of surgery that led to admissions was the trunk in about 268 (44.4%) respondents, followed by the lower limbs (n=248; 41.2%) and head and neck (n=28; 4.6%). Social-demographic features of the respondents were not significantly associated with the development of DVT (P>0.05).

Table 2 depicts various clinical variables associated with patients. A total of 218 (36.2%) patients were admitted to the orthopedic surgery unit, 146 (24.3%) to the general surgery unit, and 96 (15.9%) to urology. A large number (n=326; 54.2%) had been admitted to the emergency department. When the tests for varicose veins and clotting were conducted, 2.3% (n=14) and 0.8% (n=5) tested positive, respectively. Among the patients who had the Doppler test, about two-thirds tested positive, which was 3% (n=18) of the total respondents and both sides of the body were commonly affected with DVT in 10 (1.7%) patients after Doppler.

**Table 2:** Medical characteristics

Variables	Frequency, N=602	Percentage (%)
Surgery Unit		
Orthopedics	218	36.2
Neurosurgery	66	11.0
Cardiothoracic surgery	5	0.8
Plastic and reconstructive surgery	5	0.8
Trauma	9	1.5
General surgery	146	24.3
Urology	96	15.9
Ear, nose, and throat (ENT)	9	1.5
Other	48	8
Department Presented At		
Emergency	326	54.2
Inpatient	13	2.2
Outpatient	131	21.8
Other	132	21.9
Varicose Veins		
Yes	14	2.3
No	588	97.7
Clotting		
Yes	5	.8
No	597	99.2
Result of Doppler		
Not done	575	95.5
Positive	18	3.0
Negative	9	1.5
Deep Vein Thrombosis side		
None	583	96.8
Right	8	1.3
Left	1	.2
Bilateral	10	1.7

Table 3 depicts the association between socio-demographic characteristics and DVT. The prevalence of DVT among the respondents was 7% (n=42). The sex-specific prevalences were 6.97%(n=26) and 8.56%(n=16) for male and female, respectively. The results indicate that there was a statistically significant association between orthopedic cast and DVT (p-value=0.000). A statistically significant result was also found between Doppler test and DVT (p-value =0.032). The other variables like gender, age group, occupation, and education did not show statistical significance (p-value> 0.05).

**Table 3:** Relationship between socio-demographic/medical characteristics and DVT The data has been presented as frequencies (n), chi-square test (ꭓ2), and p-values. ** Not significant P>0.05; *significant P<0.05; ^ a^ Fisher's exact test DVT: deep vein thrombosis

Socio-demographic features	DVT		Test statistics ꭓ2	p-value
	Yes	No		
Gender				
Male	26	373		
Female	16	187	0.387	0.534**
Age group				
< 50 years	20	330		
≥ 50 years	22	230	2.053	0.152**
Occupation				
Unemployed	19	200		
Employed	23	360	1.531^a^	0.216**
Education				
High school and less	33	370		
Bachelor’s degree and more	9	189	2.711	0.100**
Marital Status				
Married	27	354		
Not married	15	206	.019	.889**
Department Presented				
Emergency	22	304		
Others	20	256	0.057	.811**
Site of Surgery				
Limbs	23	261		
Head and neck	1	27		
Thorax and abdomen	18	272	-	0.518**
Orthopedic Cast				
Yes	3	181		
No	39	379	-	.000a
Able to Get Up From Bed and Mobile				
Yes	17	214		
No	25	346	0.085	0.771**
Long Flights				
Yes	6	69		
No	36	491	-	.633a
Doppler				
Not done	38	537	8.808^a^	032*
Positive DVT	4	14		
Negative DVT	0	9		

Table 4 shows the results of a bivariate regression. The significant results were obesity (OR=17.9, 95%CI=5.39-59.18), hypertension (OR=0.9, 95%CI=0.42-1.93), ischemic heart disease (OR =4.5, 95%CI=1.18-16.83), orthopedic cast (OR=0.1, 95%CI=0.013-0.240), and the use of Doppler ultrasound (OR=0.018, 95%CI=0.00-0.92).

**Table 4:** Bivariate logistic regression analysis showing risk factors of DVT The data has been presented as frequencies (n), odds ratio (OR), and p-values *P <0.05 significant; **P<0.05 not significant. DVT: deep vein thrombosis

Risk Factors	DVT Positive	DVT Negative	Crude OR, 95%(CI)	Adjusted OR, 95%(CI)	p-value
Obesity					
Yes	9	16			
No	33	544	9.3 (3.81-22.56)	17.9 (5.39-59.18)	0.00*
Diabetes					
Yes	18	163			
No	24	397	1.8 (.965-3.46)	1.2 (.41-3.56)	.733**
Hypertension					
Yes	9	130			
No	33	430	0.9 (.42-1.93)	0.3 (.08-1.03)	0.05*
Ischemic Heart Disease					
Yes	6	49			
No	36	511	1.7 (.698-4.33)	4.5 (1.18-16.83)	0.03*
Orthopedic Cast					
Yes	3	181			
No	39	379	0.2 (.05-.53)	0.1 (.013 -.240)	0.00*
Get Up From Bed					
Yes	17	214			
No	25	346	1.1 (.58-2.08)	1.6 (0.60-4.09)	.350
Doppler					
Yes	4	22			
No	38	538	2.6 (.844-7.85)	0.018(0.00-.92)	.045*
Contraceptive					
Yes	4	43			
No	38	517	1.3 (0.43-3.71)	2.5 (0.58-10.32)	.222**
Pregnancy					
Yes	1	13			
No	41	547	1 (0.131-8.04)	0.194 (0.09-4.19)	.296**
Heparin Use					
Yes	24	236			
No	18	324	1.8 (0.97-3.51)	2.04 (0.46-9.02)	.346**

## Discussion

The prevalence of DVT among the respondents was 7% (n=42). However, the sex-specific prevalences were 6.97%(n=26) and 8.56%(n=16) for males and females, respectively. This prevalence aligns with a few studies conducted in Saudi Arabia [8,9]. However, the incidence of DVT varies greatly among general populations. The disparities in prevalence could be caused by inconsistencies in diagnostic approaches in DVT such as the use of Doppler ultrasound or venography and differences in clinical manifestations [8].

After evaluating the prevalence of DVT, it was imperative to explore its risk factors. The current study revealed that there was a statistically significant association between obesity and DVT (p-value=0.000). Obese respondents were nearly 18 times (OR =17.9) more likely to develop DVT than the non-obese respondents. Excess body fat can lead to chronic inflammation, which in turn promotes the formation of blood clots. Additionally, obesity is often associated with a sedentary lifestyle, which reduces blood flow and increases the likelihood of blood pooling in the veins, creating an environment conducive to clot formation [9]. Various studies have revealed different predictors for DVT. A study conducted among the adult population in Al-Madinah province in Saudi Arabia revealed obesity and immobility as the main risk factors. The study further found that immobility caused by orthopedic casts increased the risk of DVT [10]. These results are consistent with the findings from our study.

This study further revealed that patients with ischemic heart disease had a higher chance of developing DVT than those without (adjusted OR (aOR)=4.5). Previous studies have shown a relationship between ischemic heart disease. According to Alkarithi et al., ischemic heart disease may trigger plaques, which can lead to clots in the leg or the pelvis veins, hence increasing the risk of DVT [11]. Hypertension is another risk factor for DVT. Our results reveal that hypertensive respondents are 0.3 more likely to have DVT compared to non-hypertensive respondents. A Study conducted at King Abdulaziz University, Jeddah, Saudi Arabia, revealed that hypertensive people were more susceptible to DVT [12].

A statistically significant relationship was found between the site of surgery and DVT (p-value <0.05). This suggests that the location of surgery is a predictor of the development of DVT. Certain types of surgery, especially those involving the lower extremities or pelvis, can increase the risk of blood clot formation [12]. Similarly, an orthopedic cast and the use of Doppler ultrasound showed a statistically significant result (p-value= 0.000 and 0.032, respectively). Many studies have asserted that age and sex are predictors of DVT [13-15]. However, the current study did not find any statistically significant relationship between these two variables. This could be attributed to heterogeneity as the sample is highly diverse in terms of age, hence difficult to detect significant differences.

This study had a few limitations. First, this research was conducted solely at Aseer Central Hospital, which may limit the generalizability of the results to a broader population. Variations in patient demographics, surgical procedures, and healthcare practices in other settings could yield different prevalence rates and risk factor associations. 

## Conclusions

The prevalence of DVT in the study population was found to be significantly low, a figure consistent with previous research conducted in Saudi Arabia. Notably, the study identified significant risk factors associated with DVT, including obesity, ischemic heart disease, and hypertension. Obese individuals were nearly 18 times more likely to develop DVT than non-obese individuals, emphasizing the role of excess body fat and sedentary lifestyles in promoting clot formation. Additionally, the study highlighted the association between ischemic heart disease and DVT, potentially linked to plaque formation triggering clot development. Furthermore, surgery location, orthopedic cast, and the use of Doppler ultrasound were found to be significant factors influencing DVT risk. Surprisingly, age and sex were not identified as significant predictors in this study. These findings underscore the importance of recognizing and addressing these risk factors in clinical practice to improve the early prevention and treatment of DVT among adult surgical patients.
